# A usability study in patients with stroke using MERLIN, a robotic system based on serious games for upper limb rehabilitation in the home setting

**DOI:** 10.1186/s12984-021-00837-z

**Published:** 2021-02-23

**Authors:** Silvia Guillén-Climent, Ainara Garzo, María Nieves Muñoz-Alcaraz, Pablo Casado-Adam, Javier Arcas-Ruiz-Ruano, Manuela Mejías-Ruiz, Fernando Jesús Mayordomo-Riera

**Affiliations:** 1 Maimonides Biomedical Research Institute of Cordoba (IMIBIC), Reina Sofia University Hospital, University of Cordoba, Córdoba, Spain; 2 Neurorehabilitation area, Health Division of TECNALIA, Basque Research and Technology Alliance (BRTA), San Sebastián, Spain; 3 Interlevel Clinical Management Unit of Physical Medicine and Rehabilitation, Reina Sofía University Hospital of Córdoba, Córdoba, Spain; 4 Córdoba and Guadalquivir Health District, Andalusia Health Service, Córdoba, Spain; 5 Department of Applied Physics, Radiology and Physical Medicine, University of Córdoba, Córdoba, Spain

**Keywords:** Stroke, Neurological rehabilitation, Upper extremity, Telerehabilitation, Serious games, Home training, Robot

## Abstract

**Background:**

Neuroscience and neurotechnology are transforming stroke rehabilitation. Robotic devices, in addition to telerehabilitation, are increasingly being used to train the upper limbs after stroke, and their use at home allows us to extend institutional rehabilitation by increasing and prolonging therapy. The aim of this study is to assess the usability of the MERLIN robotic system based on serious games for upper limb rehabilitation in people with stroke in the home environment.

**Methods:**

9 participants with a stroke in three different stages of recovery (subacute, short-term chronic and long-term chronic) with impaired arm/hand function, were recruited to use the MERLIN system for 3 weeks: 1 week training at the Maimonides Biomedical Research Institute of Cordoba (IMIBIC), and 2 weeks at the patients’ homes. To evaluate usability, the System Usability Scale (SUS), Adapted Intrinsic Motivation Inventory (IMI), Quebec User Evaluation of Satisfaction with assistive Technology (QUEST), and the ArmAssist Usability Assessment Questionnaire were used in the post-intervention. Clinical outcomes for upper limb motor function were assessed pre- and post-intervention.

**Results:**

9 patients participated in and completed the study. The usability assessment reported a high level of satisfaction: mean SUS score 71.94 % (SD = 16.38), mean QUEST scale 3.81 (SD = 0.38), and mean Adapted IMI score 6.12 (SD = 1.36). The results of the ArmAssist Questionnaire showed an average of 6 out of 7, which indicates that MERLIN is extremely intuitive, easy to learn and easy to use. Regarding clinical assessment, the Fugl-Meyer scores showed moderate improvements from pre- to post-intervention in the total score of motor function (p = 0.002). There were no significant changes in the Modified Ashworth scale outcomes (p = 0.169).

**Conclusions:**

This usability study indicates that home-based rehabilitation for upper limbs with the MERLIN system is safe, useful, feasible and motivating. Telerehabilitation constitutes a major step forward in the use of intensive rehabilitation at home.

*Trial registration* ClinicalTrials.gov, NCT04405609. Registered 06 January 2020—Retrospectively registered, https://clinicaltrials.gov/ct2/show/NCT04405609

## Background

Strokes are among the leading causes of death, physical disability and economic burden worldwide [1, 2]. The prevalence of people living with the effects of stroke has increased over the last few years, thus creating a higher demand for rehabilitation services [3]. The paralysis of the upper limbs is a common impairment after strokes, and only 10–20% of patients recover completely [4, 5]: for these patients, the main aim of arm rehabilitation is to recover lost functions [6]. Nowadays, the key aspects to make rehabilitation effective for people with stroke are considered to be intensity, repetition and using suitably challenging and function-oriented activities [7–9]. However, the increase in the number of people affected and the current limitation of health resources make it very difficult to provide services using a traditional approach.

Continuous advances in neuroscience and neurotechnology are transforming stroke rehabilitation [10]. At a time when the rehabilitation services resources are unable to meet the demand, robot-assisted rehabilitation and home-based telerehabilitation are gaining greater acceptance [11]. Robot-based neurorehabilitation systems provide a solution to increase the number of movements, involve safe, intensive rehabilitation exercises [12, 13] and have the advantage that the precise movements of the robot are able to measure the patients’ movements objectively [14, 15]. On the other hand, home-based telerehabilitation allows us to extend institutional rehabilitation by increasing and prolonging the therapy [16]. What is more, the combination of game-based telerehabilitation and robotic systems creates a motivating, engaging environment for patients [17]. The enjoyment patients derive from playing these so called ‘serious games’, designed specifically for the rehabilitation tasks, can greatly increase the quality and quantity of the therapy delivered [18].

MERLIN is a robotic system based on serious games for the upper limb tele rehabilitation in patients with a stroke. It is presented as an affordable and easy to use solution to allow the patient to carry out an intensive rehabilitation at home, with a continuous remote monitoring and communication with the therapist. The system is composed of an upper-limb rehabilitation robot and a software platform which guides and measures the patient’s movements and allows physicians to customize the therapeutic plan and to monitor the patients’ evolution.

The purpose of this manuscript is to present the usability validation of MERLIN system. In this study, we evaluate the ease to use, consistency and acceptance of the system have been evaluated. The research carried out also aims to demonstrate the feasibility of including the robotic therapy as a complement to a regular daily rehabilitation program.

## Methods

### Participants

In order to detect most problems of usability which can affect a product, Jakob Nielsen’s theory [19] regarding the sufficient number of users to evaluate a system is widely accepted. According to Nielsen, between three and five users can identify 85% of the most relevant usability problems. In this case, due to the heterogeneity of the study population, it was decided to recruit 12 patients at different stages of post-stroke upper limb recovery, in order to test as many system features as possible.

 Participants were recruited at the Reina Sofía University Hospital in Cordoba, Spain. The participants were divided in three different groups, depending on their stage of recovery: subacute (2–6 months of recovery of stroke), short-term chronic (6–12 months) and long-term chronic (over 12 months). Four patients were recruited from each stage. The inclusion criteria to participate in the study were: subjects over 18 with upper limb hemiparesis after stroke, unilateral paresis and cognitive ability to understand, accept and actively participate in the usability study. Having Wi-Fi at home and a table measuring 110 × 68 cm on which the MERLIN system can be set up was considered also a requirement to participate in the study. Patients who presented bilateral motor deficit, severe spasticity, psychiatric illness, and/or cognitive impairment were excluded.

 All the subjects were duly informed about the study and all of them gave their written consent before the first session.

### Study design

This interventional study is an open label trial with a single group and a longitudinal design. Each patient used the MERLIN system for 3 weeks: 1 week training at the IMIBIC (Maimonides Biomedical Research Institute in Córdoba, Spain) with the supervision of a physiotherapist, 1 week at the patient’s home with similar supervision and 1 week at patient’s home on their own with remote support and supervision of a physiotherapist to organize the rehabilitation sessions.

Arm and hand functions were evaluated at baseline (on day 1 before starting the training), and on the last day. The usability of the system and the participants’ motivation was evaluated on the last day using different validated scales, as explained below.

### MERLIN unactuated robotic telerehabilitation system

The MERLIN system has been developed to bring neurorehabilitation to the post-stroke patients’ homes with the aim of providing daily, intensive, motivating and patient-tailored rehabilitation, with the indirect supervision of the therapist [20]. The system is composed of ArmAssist (AA), a cost-effective robotic system based on serious games developed by TECNALIA, and the Antari Home Care platform [21] to supervise, organize and customize the patients’ daily training remotely, which has been developed by GMV [22]. The AA system is a modular solution which includes an affordable, portable robotic device for a complete upper limb rehabilitation, and a software platform based on serious games to motivate the patients and assess their training [18].

**Fig. 1:**
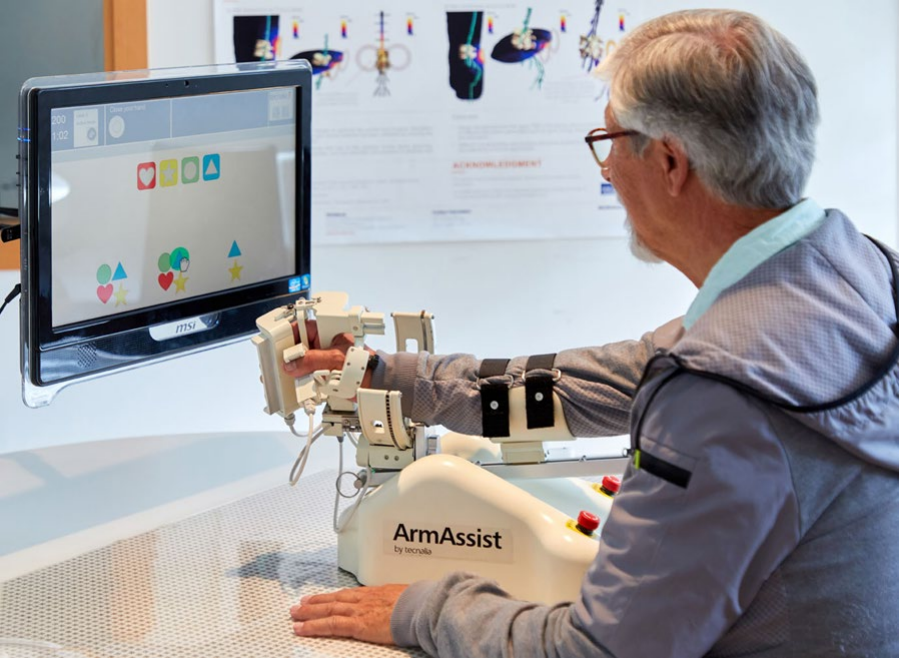
AA system

In the MERLIN system, the non-actuated version of the AA robotic device has been used to ensure a safe use in the home environment when continuous supervision is not feasible. The AA device includes several sensors to measure the patient’s active self-directed active movements during the games, which are performed on a normal table to control the games (see Fig. 1). The device can be easily fastened on the forearm, and allows natural movements with low resistance. The position and orientation of the robot are calculated using the information from the camera, which reads the QR codes on the mat below, and the encoders included on the wheels. Wrist angle, hand grasping force and vertical arm force are calculated by a potentiometer, and two Force Sensing Resistors (FSR) and a load cell are included on the hand module and arm support, respectively. The key movements that can be measured are: three types of movements over the table, horizontal shoulder abduction-adduction, flexion–extension in the elbow (vertical force), wrist prono-supination movements and hand opening and closing [23]. This version of the system is aimed at patients who can actively carry out the movements and is thus more appropriate for patients who have mild or moderate motor impairment according to the Fugl-Meyer scale, who are more suited to continuing the therapy at home. The movements are used to interact with the implemented serious games on the software, which are divided into different levels depending on the patients’ stage of recovery and cognitive capabilities. The games include assessment and training [24] and they were co-designed by patients and physiotherapists [25]. 7 training games are available, such as choosing letters to make a word, discovering pairs, solitaire or doing a puzzle, for example. Additionally, the option of using some online games is also available. This option is recommended for patients with good movement control and cognitive capabilities. The games can be configured for only some movements or a combination of different ones. The exercises involve extending the user’s range of exercises beyond their normal threshold, which has been previously set by the assessment games, and can be modified when necessary, i.e. when motor improvement is detected by the physiotherapist. The games have been adapted to for the target group taking into account any possible cognitive or visual problems [26].

**Fig. 2:**
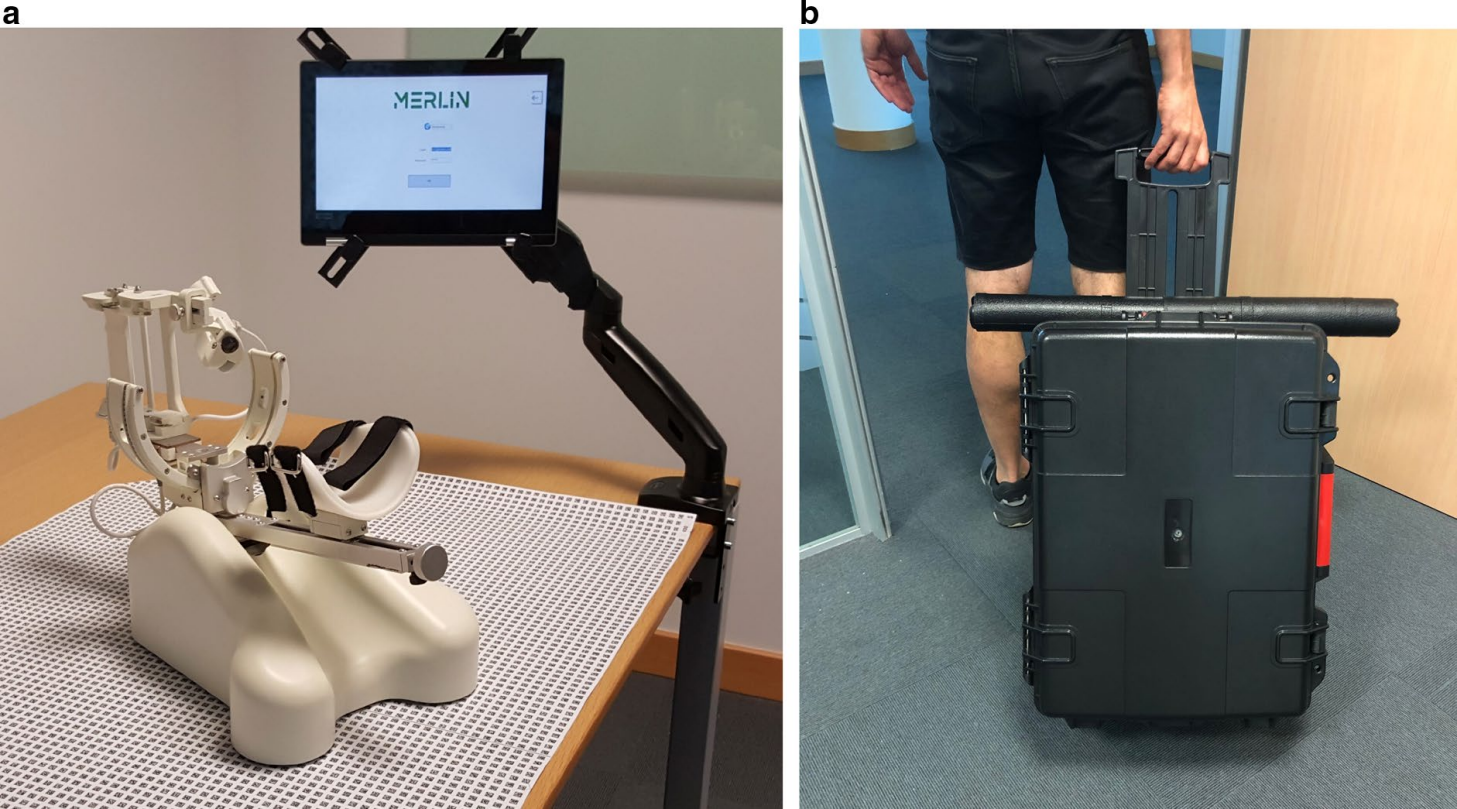
Adaptations made to the system for home use. **a** Adaptation of mat. **b** Package

With the MERLIN system, the patients can access the daily therapy previously organized by the therapist, as well as viewing a summary of the results obtained during therapy (see Fig. 2). It also features a messaging tool to communicate with the therapist, similar to mailing.

**Fig. 3:**
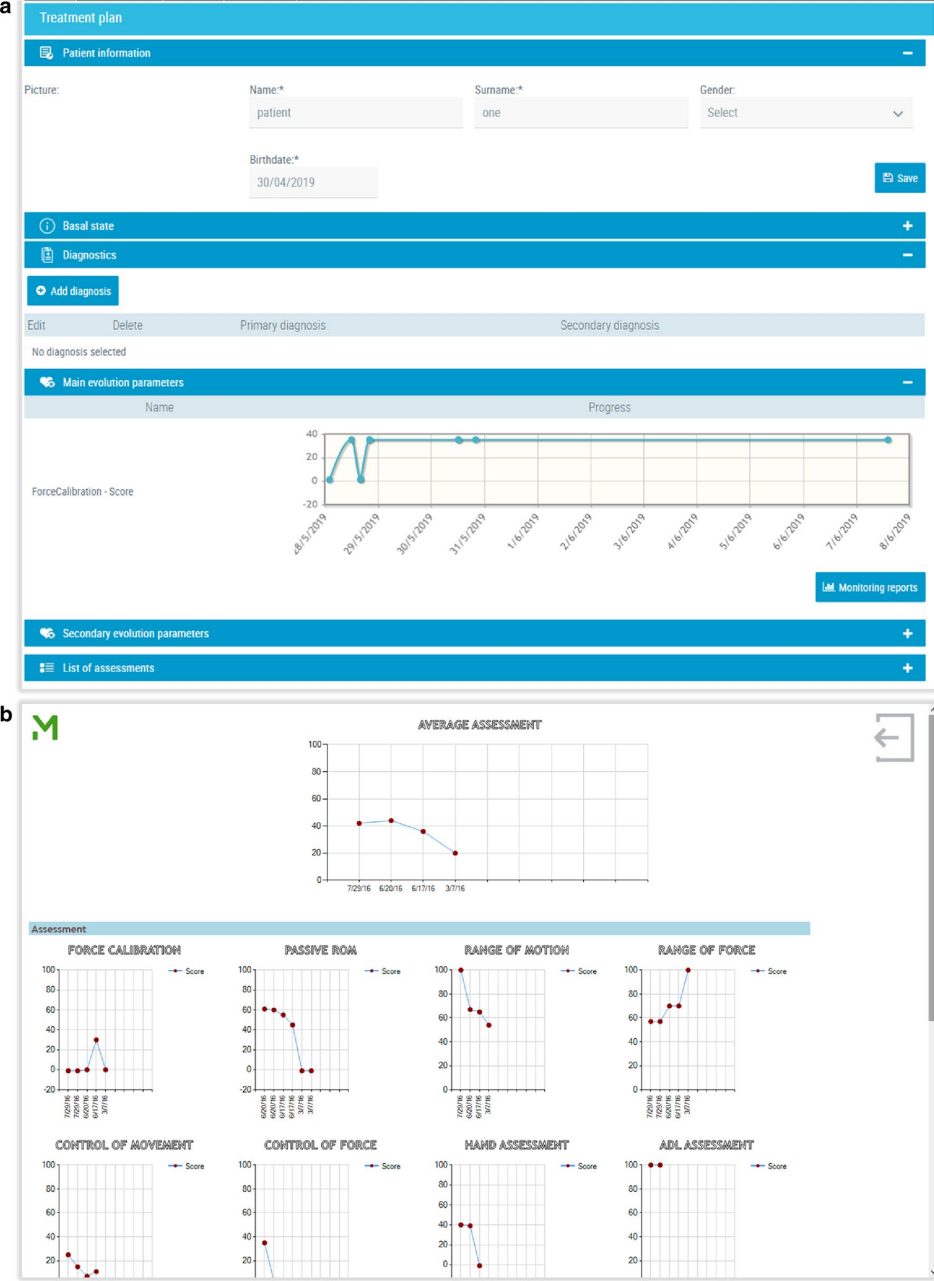
MERLIN system. **a** Results of the therapy shown on the therapist’s panel. **b** Evolution of the therapy shown on the patient’s panel

The AA system has been previously tested in a clinical setting by therapists and patients with positive results of acceptance [11] and effectiveness, with improvements in the patients’ motor function after use [24]. Previous studies have also demonstrated that the therapy using serious games and the AA system is enjoyable and motivating because the patients feel more engaged [27]. In the present study, the system was adapted for home use using the non-actuated version of the robot for greater safety. With this aim, the software was programmed to work on a tablet to make the system more compact and adaptable to the home-setting and package was designed to transport the system, and the mat was adapted to make the system easy to transport and store (see Fig. 3).

As previously explained, the Antari HomeCare platform has also been integrated into the MERLIN system. This telecare platform designed for managing patients’ treatments and online follow-up, was adapted for remote customizing of rehabilitation therapies. The therapist used the online platform to plan each patient’s therapy and selected the games to be used, the movements to train, the number of days to be repeated and the length of each game. Monitoring the patient’s evolution and therapy (duration of training, frequency, points obtained, etc.) was performed online using this software (see Fig. 2). The messaging tool was accessible via the Antari HomeCare system.

The aim of this usability study was to evaluate the users’ acceptance of the new features and remote monitoring carried out by the therapist, instead of the face-to-face monitoring usually performed in previous evaluations of AA [11, 24, 27]. The system safety when patients work independently at home was also evaluated, and this feedback on usability, acceptance, motivation and safety is an important input for demonstrating how easy the system is to learn and use.

### Intervention sessions

**Fig. 4:**
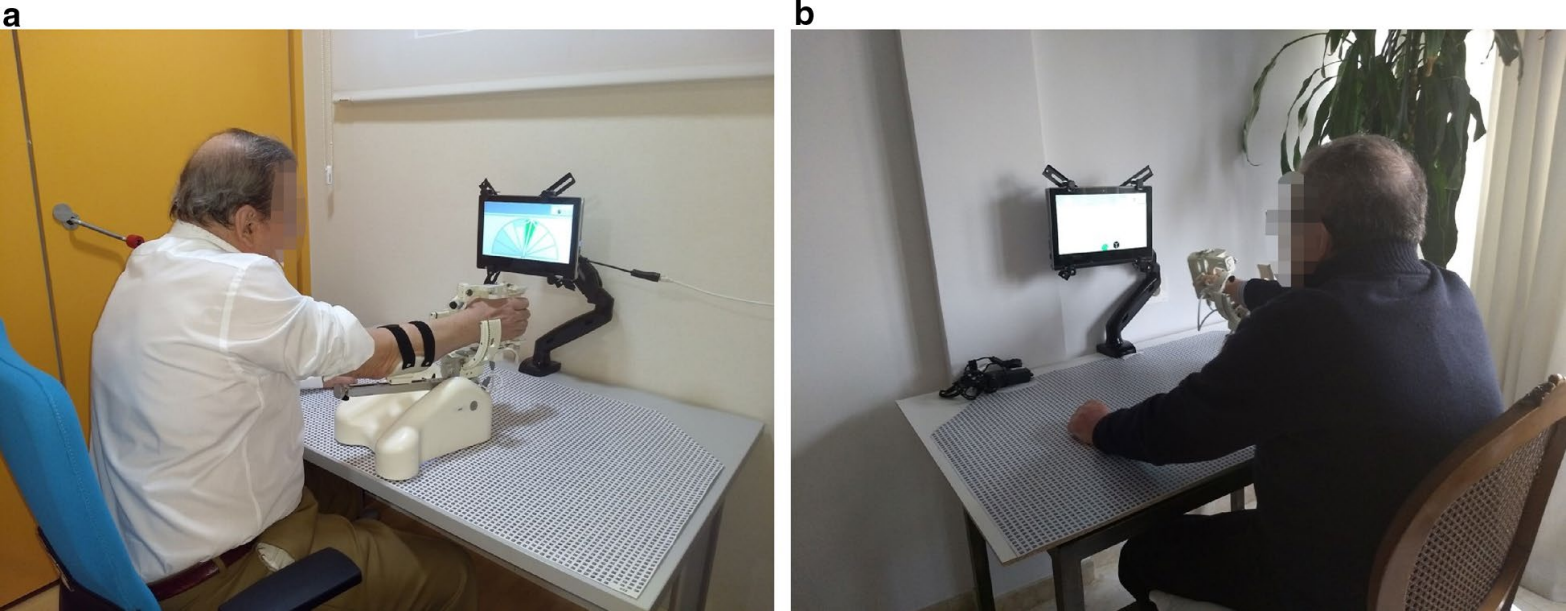
Usability of study pictures. **a** Session at IMIBIC facilities. **b** Session in a home environment

Rehabilitation therapy included 11 sessions using the MERLIN system performed over a period of 3 weeks. The first week was used as training to teach users and caregivers how to use the system correctly, as well as for getting used to the rehabilitation system, robot movements and protocol times. The training sessions were organized every day for 1 h at the IMIBIC facilities (see Fig. 4a). Special emphasis was placed on the correct positioning of the arm and shoulder for rehabilitation. In addition, each participant received a copy of the user’s manual, which also included a telephone number to contact in case of any technical or clinical problems. The therapist installed the system at the participant’s home at the beginning of the second week, adjusted the chair height and explained to the patient the correct back and shoulder positions for doing the training as well as the most comfortable arm position for rehabilitation when supervision was not available (see Fig. 4b). 3 sessions of 30 min were held on alternate days. During this week, the patient carried out the therapy assigned for each day with the supervision of the therapist. The third week followed the same timetable, except that the participants were trained to use the system at home completely autonomously. The therapist used the tele-care platform daily to follow up remotely the participants’ progress and their use of the system, as well as for organizing the following sessions.

The training movements and games used for this purpose were selected by the therapist, who decided on the intensity level and movements to train in the therapy according to the patient’s evolution or cognitive conditions. The therapist organized the rehabilitation sessions beforehand, using the tele-care platform designed for this purpose. Prior to commencing therapy, patients were requested to perform a calibration process to set up the threshold according to their range of motion. This allowed the participants to set the level of challenge in the exercises at their maximum capacity. After that, while using the system, the range of motion for each game and patient was controlled by the system itself.

### Assessment

The study data were collected and managed using REDCap [28] electronic tool hosted at FIBICO (Foundation for Biomedical Research in Córdoba, Spain) [29]. REDCap (Research Electronic Data Capture) is a secure, web-based software platform designed to support data capture for research studies, providing (1) an intuitive interface for validated data capture; (2) audit trails for tracking data manipulation and export procedures; (3) automated export procedures for seamless data downloads to common statistical packages; and (4) procedures for data integration and interoperability with external sources [30, 31]. REDCap is HIPAA (Health Insurance Portability and Accountability Act) [32] and 21 CFR Part 11 [33] compliant, which means that it meets the minimum level of security for data in clinical investigations. However, no personal data were recorded on REDCap, in compliance with the European General Data Protection Regulation (GDPR) [34], as the participants were European citizens.

#### Primary outcomes measurements. Usability and acceptance data

The feasibility of use of the system and motivation were evaluated by the patients using semi-structured interviews and different usability questionnaires with Likert scales during the clinical trials, which lasted for 3 weeks. The validated scales used were: the System Usability Scale (SUS) [35], the Adapted Intrinsic Motivation Inventory (IMI adapted) [36], the Quebec User Evaluation of Satisfaction with assistive Technology (QUEST) [37], and the AA Usability Assessment Questionnaire [18].

The SUS scale was used to evaluate the usability of the system. The scores ranged from 0 to 100%, where a higher score means better usability, with a threshold of 68%.

The IMI scale is a multidimensional measurement method designed to assess participants’ subjective experience related to a given activity. The full version of the questionnaire includes 45 items and 7 subscales. Each item is used to rate the statement on a scale ranging from 1 ‘strongly agree’ to 7 ‘strongly disagree’ [36, 37]. In accordance with the self-determination theory, this scale allows the researcher to decide which items to use in order to create a shorter version of the questionnaire [36]. The version used in the current study consisted of twenty items divided into six subscales: interest/enjoyment, perceived competence, effort, pressure/tension, perceived choice and value/usefulness. The version used can be found in the complementary documentation (see Additional file 1) and is very similar to the IMI questionnaires used in other usability studies with robotic devices [38, 39].

The purpose of QUEST is to evaluate the patient’s satisfaction with the device and with the services they have used. It consists of 12 questions: 8 related to the device and 4 related to the services, which must be rated on a Likert scale from 1 ‘Not satisfied at all’ to 5 ‘Very satisfied’. The AA Usability Assessment Questionnaire consists of a 17-item survey and was specifically designed for the AA device used in MERLIN system. The questions are rated by patients and therapists from 1 ‘strongly agree’ to 7 ‘strongly disagree’ to evaluate the satisfaction with the system and the therapy. It also includes three open-ended questions about the participant’s subjective opinion, such as the aspects liked most, any negative aspects identified, and any proposals for improving the system.

In addition, two short questions were added to ask the participants about their willingness to pay for the MERLIN system as therapy.

#### Secondary outcome measurements. Clinical information

With the aim of quantifying general arm function and any effects the system has on it, clinical standardized scales were used, before the patients started with the therapy using MERLIN and after finishing the clinical trial. The Fugl Meyer Upper Extremity Assessment Scale (Fugl-Meyer) [40] and the Modified Ashworth Scale (MAS) [41] were used to evaluate the patients’ clinical condition before their enrolment in the study to confirm their participation according to the inclusion criteria. The same scales were repeated at the end of the therapy using MERLIN to confirm the safety of the system and that no negative effects had been caused in the patients such as reduction of arm function. Fugl-Meyer and MAS could also be used to measure the effectiveness of the system, although only small improvements were expected due to the short duration of the intervention, in which only limited clinical evidence could be obtained.

The Fugl-Meyer is an index to assess the sensorimotor impairment in individuals. The MAS measures muscle tone during passive soft tissue stretching by rotating a joint and estimating the resistance, and it is used as a simple measure of spasticity.

### Statistical analysis

The statistical outcomes were analysed using IBM SPSS Statistics© [42] software for Windows© Operating System. Descriptive summary statistics (mean with standard deviation, SD) was used to process the quantitative data provided by the Likert-scale items in SUS, QUEST and Adapted IMI. The qualitative data obtained in the open-ended questions were analysed using thematic analysis.

For the clinical assessment, a one-tailed paired t-test with a significance level of p < 0.05 was used to compare pre- and post-intervention Fugl Meyer and MAS outcome measures.

## Results

### Participants

This clinical study was planned to start in September 2019 and finish in June 2020. During the study, the COVID-19 global pandemic broke out, which had a significant impact in the study. It was foreseen that 12 patients with a post-stroke hemiparesis would participate in the research, but only 9 of these completed the intervention study. 3 patients dropped out of the study after the recruitment period due to the pandemic. A special effort was made to recruit additional participants to complete the study, but no patients were willing to participate in the study during the research period. The patients with a post-stroke upper limb impairment included in this research usually had a comorbidity such as hypertension, mellitus diabetes, atherosclerosis, heart disease, etc. which includes this population in the risk group for COVID-19. We were fully aware of this situation, as well as the mobility restrictions imposed by the government, and because of fear of infection, it was not possible to include additional participants at the end of the study. However, it was considered that the results are also reliable with 9 patients, as was previously explained.

66.7% of the participants were men and 33.3% women. Their age range was between 41 and 89, with an average of 63.9. 66.7% had left hemiparesis and 33.3% right.

In addition to the therapy received with the MERLIN system during the usability study, 88.9% of the patients also received other Occupational Therapy or Physiotherapy sessions (public or private). The characteristics of the nine participants are shown in Table 1.

**Table 1:** Demographic information of patients

	Age	Gender	Hemiparesis	Dominant Hand	Months since stroke	Employment situation	Other therapies
P1	59	M	L	R	2	Medical leave	Y
P2	60	M	R	R	5	Medical leave	Y
P3	70	F	L	R	6	Retired	Y
P4	74	M	R	R	6	Retired	Y
P5	41	M	L	R	37	Medical leave	Y
P6	42	F	L	R	17	Unemployed	Y
P7	89	M	L	R	4	Retired	N
P8	69	F	L	R	7	Retired	Y
P9	71	M	R	R	35	Retired	Y

Gender: M = Male, F = Female; Hemiparesis/Dominant Hand: L = Left, R = Right; Other therapies: Y = Yes, N = No

The participants were also asked at the end of each session about any adverse effects that may have happened during the clinical trial: no relevant adverse effects were reported. The participants reported only two drawbacks that have been previously foreseen in the user’s manual: (1) chafing on the skin due to contact with the robot’s protruding elements; and (2) mild shoulder fatigue at the end of the session. To avoid any chafing, strips of foam were provided to attach to any parts of the robot that could cause the problem. This solution should be customized to the participant’s arm/hand size. As regards shoulder fatigue, the timing of the sessions was adjusted to 20–30 min for all the participants with the aim of avoiding this adverse side effect.

### Usability and acceptance of results

**Fig. 5:**
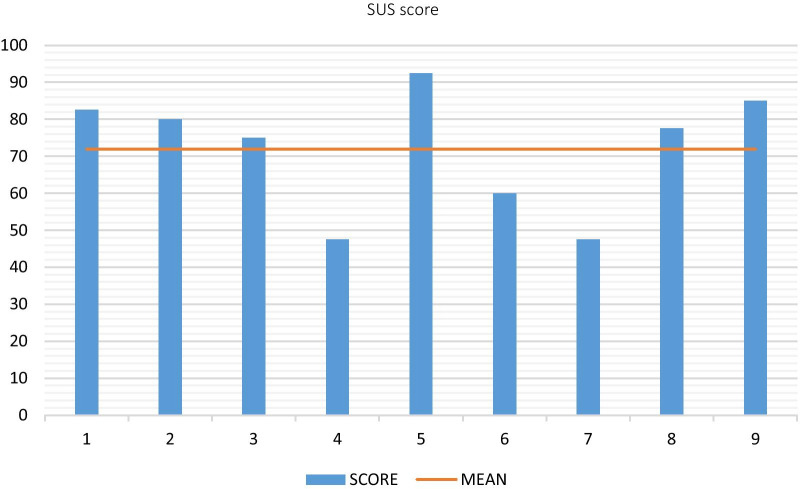
Individual results on SUS Scale (0–100 %)

The quantitative data obtained using SUS, QUEST and IMI questionnaires is summarized in Figures below, which show the results of user acceptance and experience. The usability perception has been rated with a mean score of 71.94 % (SD = 16.38) on the SUS scale (see Fig. 5), which means that the system usability is considered “Good”, according to the research by Bangor et al. [43]

**Fig. 6:**
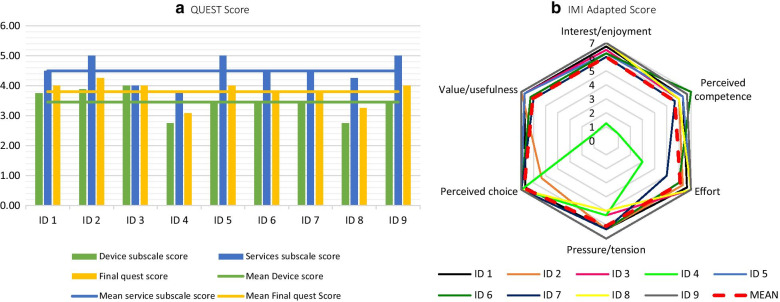
Individual results. **a** QUEST Scale. **b** IMI Adapted Scale

According to the IMI (see Fig. 6b), the participants considered the intervention useful (6.06 ± 1.93), considered themselves competent (5.67 ± 1.79), did not feel pressurized (6.11 ± 0.73) and reported high levels of interest and enjoyment (6.00 ± 1.82). They also agreed that they had participated voluntarily (6.78 ± 0.55) and evaluated their own effort positively (6.11 ± 1.34). The motivation and satisfaction levels were positive, as reflected in a mean score on the QUEST scale of 3.81 out of 5 (SD = 0.38) (see Fig. 6a). On both those scales, a higher score means that the participant is more motivated or satisfied (the result of the pressure/tension subscale has been normalized).

The results of the AA Questionnaire test are presented in Table 2. The participants rated the system with an average of 6 on a scale of 7, and it can therefore be concluded that they considered it easy to learn, easy to use and intuitive. They also considered that the system affects their treatment positively because it allows them to train longer, and they reported that this therapy could be more entertaining compared to the regular therapy (6 out of 7). All the participants agreed that they would recommend the system to other patients, but some improvements were proposed. Some examples of the participants’ proposals can be found in the next section (see Open-ended questions).

**Table 2:** Patients’ results from the AA questionnaire

Questions		P1	P2	P3	P4	P5	P6	P7	P8	P9	MEAN
1. It has been easy to learn how to use the system, both the hardware and the software		7	7	7	5	7	7	3	7	7	6.33
2. I think I will often need the support of a technical person to be able to use this system		2	4	5	5	1	1	6	2	6	3.56
3. Using this system, I need to spend a lot of time in non-training activities		2	2	4	2	1	4	2	1	1	2.11
4. I can remember without problem how to use the system effectively		7	7	4	3	7	7	3	7	7	5.78
5. It took a long time to be able to use the system without problems		2	7	2	1	1	1	1	1	1	1.89
6. I think that I will benefit from using this system		7	7	7	1	7	7	7	7	7	6.33
7. Using this system I am motivated to train longer		7	6	7	1	5	7	4	7	7	5.67
8. I think that this system is uncomfortable to use		5	3	5	7	4	5	4	7	2	4.67
9. I enjoyed training with this system		7	6	7	1	5	7	5	7	7	5.78
10. I would recommend other people to use this system		7	7	6	4	7	7	6	7	7	6.44
11. I think that this system needs to be improved		7	6	6	7	7	5	4	7	6	6.11
12. I had internet connection problems while using the system		4	2	5	6	1	3	2	6	1	3.33
13. I feel uncomfortable using a system like this, because I have no experience in using a pc		1	1	4	1	1	1	6	1	2	2.00
14. I don’t think using this system will make any change to my condition		2	1	2	7	1	1	5	1	2	2.44
15. I feel that the games are inadequate for the training		2	1	1	4	6	1	1	1	2	2.11
16. I am familiar with this kind of technology		6	4	2	2	4	4	1	7	2	3.56
17. I feel myself safe using this system		6	7	6	2	7	7	7	7	6	6.11

On the scale, 1 indicates ‘Strongly disagree’ and 7 ‘Strongly agree’

#### Open-ended question

The participants were motivated to participate both during the clinical trials and the rehabilitation sessions. At the end of the trials, all of them gave positive answers to the questions regarding the use of the MERLIN system at home. The patients stated that they had enjoyed their participation and valued positively this different style of therapy for the rehabilitation of their affected arm. Some of them stressed the aspects they liked most:

- *“It is a new attractive and motivating therapy”*.
- *“The MERLIN system is an entertaining and easy to use therapy, which allows you to repeat an exercise many times”*.
- *“We can decide on the most convenient time for doing the therapy”.*

Some participants also expressed negative aspects. The height and size of the robot did not allow all the patients to adopt a completely relaxed posture. Regarding the serious games, some participants complained about the limited range of games and how simple they were. The participants, both patients and clinicians, proposed the following future improvements:

- Improve robot design to allow a relaxed arm position.
- Adjust the dimensions and reduce the size and the area to better suit home use.
- Include more games and make them more attractive.
- Develop more complex games with greater cognitive involvement.

Finally, the participants were asked about their willingness to pay for a therapy using the MERLIN system and what price they would be willing to pay. 88.9% of the participants said they were in favour of paying for a system like MERLIN for more than 6 weeks and they would be willing to rent it for 40–60 € per month.

### Clinical results

Although the aim of these trials was not to measure the effectiveness of the system, some measurements of the mobility status of the participants were also taken before and after the use of MERLIN, with the aim of detecting any unforeseen negative effects in the patients. As previously explained, the Fugl-Meyer and MAS scales were used with this aim. The outcome measurements are shown in Tables 3 and 4. From the data gathered, it can be stated that there was a visible improvement on the Fugl-Meyer scale after rehabilitation using the system (T1), with significant changes in upper limb and coordination (p = 0.008 and p = 0.004, respectively), and in the total score for motor function (p = 0.002).

**Table 3:** Fugl-Meyer Motor function outcomes

	Upper limb (36^a^)		Wrist (10^a^)		Hand (14^a^)		Coordination (6^a^)		Motor function (66^a^)	
	T0	T1	T0	T1	T0	T1	T0	T1	T0	T1
P1	31	36	9	9	14	14	5	6	58	63
P2	23	28	7	10	10	11	4	4	44	52
P3	32	36	10	10	13	14	5	6	60	66
P4	30	31	9	9	13	13	4	4	56	57
P5	24	26	3	4	8	8	3	4	38	42
P6	26	30	7	9	14	14	5	6	52	59
P7	32	32	10	10	12	12	4	5	58	59
P8	9	10	3	3	6	7	3	3	21	23
P9	32	32	9	10	12	12	3	4	56	58
MEAN	26.56	29.00	7.44	8.22	11.33	11.67	4.00	4.67	49.22	53.22
SD	7.49	7.84	2.74	2.73	2.78	2.60	0.87	1.12	12.84	13.25
T-test	t = − 3.55; p= 0.008		t = − 2.14; p=0.065		t = − 2.0; p= 0.081		t = − 4.0; p= 0.004		t = − 4.54; p= 0.002	

T0 = baseline and T1 = post-training sessions

*SD* standard deviation, *P* Participant

^a^Maximum score for each motor component of the assessment

However, since there were no significant changes in MAS outcomes (p = 0.169), this kind of robot-based therapy does not seem to influence the spasticity of the upper limb.

**Table 4:** MAS outcomes at T0–T1

	T0	T1
P1	0	0
P2	1	1
P3	1	0
P4	0	0
P5	1	1
P6	0	0
P7	0	0
P8	2	1
P9	0	0
MEAN	0.56	0.33
SD	0.73	0.50
T-test	t = 1.512; p = 0.169	

*P* participant

^a^Maximum score in assessment = 5

## Discussion

The aim of this study was to analyse the user acceptance and usability of the MERLIN system in the home environment in patients with upper limb motor impairment after a stroke. In this study, since we also aimed to observe the possible changes in the patients’ clinical condition after participating in the trials, some additional clinical measurements were also added. When new technology for rehabilitation is developed, the final users’ special needs and their mobility limitations must be taken into consideration in order to guarantee that the system design meets their real needs and requirements [44]. In addition, the usability of the system must be evaluated to build a system which is comfortable and motivating, and which patients are willing to use, in order to ensure better adherence to treatment in the future.

Numerous studies over the last few decades have shown the effectiveness and the advantages of using robotic systems for neurorehabilitation [45–47] and tele-rehabilitation in last decades [48–50]. Other studies have also demonstrated that the use of an exoskeleton with a patient-driven control strategy for rehabilitation, in which the patient plays an active role during the therapy sessions, helps to make the treatment more attractive and therefore more effective [51]. In this study, a robotic tele-rehabilitation system based on serious games was set up for testing in the post-stroke patients’ home. This is the first time that this system has been tested in a home environment, as it was previously tested in a clinical setting [24, 18, 27].

The positive results for usability obtained in this study agree with other home-based studies published in recent years [5, 52, 53]. 9 patients completed the study and their overall ratings on the different scales were positive. The mean obtained for the SUS scale was 71.94, which is considered as ‘Good’ on the usability scale [44] and is higher than other similar studies that investigated system usability for similar technology for rehabilitation, such as Nijenhuis et al., which was rated at 69.0 [54], or Radder et al. with 70.1 [55].

In addition, the mean of 6.06 out of 7 points for IMI, with a minimum of 5.67 on each subscale, indicates that participants perceived the system as an interesting and motivational system to use. The abovementioned studies also evaluated the system using this scale, obtaining a mean of 5.2 and 5.1 respectively [54, 55], which is lower than the results obtained with the MERLIN system.

Regarding the results of the QUEST and AA Questionnaires, the participants perceived this therapy as interesting and motivating, as well as simple, intuitive and easy to use. However, P4 and P8 expressed a slightly more negative view compared to the rest. P4 was not used to using new technology and felt frustrated when interacting with the computer and games. P8 has severe motor impairment, which meant much more effort was required to move the arm compared with the other participants. Despite these drawbacks, both patients decided to complete the intervention.

The open-ended questions about the participants’ subjective opinion showed that they enjoyed the therapy using the MERLIN system, which indicates the potential for robotic systems based on serious games to be used for making people with stroke more actively involved in their rehabilitation sessions. In addition, the researchers observed and perceived that the use of feedback like games’ scores or positive messages and the possibility of following up their own evolution, motivated them: the patients were very positive about this feedback option, which helped them engage better with the therapy.

It should be highlighted that the 100% of the participants would recommend using the MERLIN system to other patients, and 88.9% would use it for more than 6 weeks. All the participants reported low or no levels of stress when using MERLIN at home on their own. Despite the general positive perception, participants also considered that MERLIN would need some improvements, which will be considered when the system is developed further. The size and height of the device, as well as the inclusion of new games and improvements in the design are some examples of proposals for usability made by the participants. Although the participants recruited in the current evaluation were not cognitively affected due to the inclusion criteria, these games were designed for all types of post-stroke patients, and so not all the software was properly adapted for the participants. However, the MERLIN system also offers the option of selecting online games, but only if the therapist considers that the patient has enough mobility control to play them and not get frustrated. The participants agreed that the number of games was limited and some of them became too easy after a few sessions of training at home. Despite the fact that during the project, the number of the objectives for each game was increased (number of images, words, etc. to be achieved), we feel that, considering the feedback given and other studies using virtual reality [56, 57] or collaboration-based games [58, 59] the number of games should be extended with the help of health professionals to engage patients in longer therapies.

As stated above, the aim of this study was to evaluate the usability of the system, and, in that respect, the intervention timeline and the number of patients used were too small to fully demonstrate the effectiveness of the therapy. However, the clinical assessment showed moderate improvements in motor function in the upper limbs which agrees with results found in other studies carried out with similar robotic systems in the home environment [60, 61]. On an individual level, the nine participants were classified for motor function as mildly (6 patients), moderately (2) and severely (1) impaired on the baseline measurements using Fugl-Meyer scale. The participants achieved a notable improvement, which was evaluated using the Fugl-Meyer motor function scale after training. The intervention involved patients in the subacute, short-term chronic and long-term chronic phases, according to the inclusion criteria. More changes were expected in patients considered subacute after training, as theoretically most recovery from specific impairments occurs during the 6 first months after stroke [62, 63]. However no great differences were found in their progress. In fact, P6, a patient in the chronic phase, experienced the greatest motor change. The most significant changes were experienced by P6, P2 and P3: these patients were in different phases of evolution and had a different baseline, as can be seen in Tables 1 and 3, respectively. Therefore, in this study, no relationship was found between motor function improvements and progress, and as most of the participants were also taking part in additional therapies, the improvements cannot be ascribed exclusively to the use of the MERLIN system.

On the other hand, since there were no significative changes to MAS (p = 0.169), it can be concluded that the use of this system does not have any influence on the upper limb spasticity. This result can be considered positive because, in addition to the intrinsic factors that cause spasticity, it has been suggested that extrinsic factors (noxious triggers) may increase the spasticity. These factors could include mental stress, physical contact, anxiety, pain, muscle fatigue, muscle contractures, certain body postures, jerky movements or changes of position, among others [64], which could induce or aggravate high muscle tone and induce pain. None of the patients reported an increase in spasticity or a reduction in mobility after using the system, which demonstrates that MERLIN does not cause any noxious triggers.

According to the clinical results, none of the patients reported a reduction of the mobility or increased the spasticity after using of the system, which demonstrates that its use at least safe.

Additionally, there were no serious adverse side effects during the study. Mild shoulder pain, mild fatigue and chafing of the skin were the only adverse effects noted. Therefore, this study provides evidence that the MERLIN system may be used safely in a home setting.

Future studies might consider greater using a number of participants including a control group, and a longer duration of training. Identifying the different patients’ factors like age, sex, severity, or evolution of stroke could be also helpful with the aim of identifying the target group. In addition, incorporating additional or more complex games and wider range of movements in the MERLIN system, would probably enhance motivation, which might lead to a more effective therapy.

## Conclusions

This study demonstrates the usability of the home-based MERLIN system in patients with upper limb motor function impairment after stroke in different stages. The usability analysis showed that almost 100% of the patients who participated found the system useful, safe and motivating, and all of them achieved moderate clinical improvement in motor function, according to the average Fugl-Meyer score.

In this study, the participants carried out 8 additional hours of upper limb rehabilitation at home with an innovative approach in neurological rehabilitation, based on serious games and using intensive, repetitive, interactive, and individualized practice. The results of this study reflect that home training with the MERLIN system with the indirect supervision of a therapist could be an interesting approach that includes the most important specific features of neurorehabilitation: it is high-intensity, task-specific, goal-setting, repetitive, functional, meaningful and challenging for the patient.

Further research with a larger sample of participants, including a control group and a longer intervention, would help to explore the efficacy of the system and to identify the factors, in order to gather better results on neurorehabilitation.

The feasibility of using this low-cost, easy to learn, easy to use and easily transportable rehabilitation system might constitute a major step forward in transferring intensive rehabilitation to the home setting. Nowadays, this factor is of special importance due to impact of the COVID-19 pandemic.

## Supplementary Information

**Additional file 1.** Intrinsic Motivation Inventory (IMI).

## Data Availability

The datasets used and analysed during the current study are available from the uicec@imibic.org on reasonable request.

## Abbreviations

AA: ArmAssist
ADL: Activities of Daily Living
ARAT: Action Research Arm Test
CFR: Code of Federal Regulations
FIBICO: Foundation for Biomedical Research in Cordoba; Fundación para la Investigación Biomédica de Cordoba in Spanish
FSR: Force Sensing Resistors
Fugl-Meyer: Upper Limb Fugl Meyer Scale
GDPR: General Data Protection Regulation
HIPPA: Health Insurance Portability and Accountability Act
IMI: Intrinsic Motivation Inventory
IMIBIC: Maimonides Biomedical Research Institute (Instituto Maimónides de Investigación Biomédica, in Spanish)
MAS: Modified Ashworth Scale
P: Participant
QR: Quick response
QUEST: Quebec User Evaluation of Satisfaction with assistive Technology
REDCap: Research Electronic Data Capture
SD: Standard deviation
SUS: System Usability Scale
T0: Evaluation measurement pre-intervention
T1: Evaluation measurement at post-training sessions
