# Supplementary material for: A usability study in patients with stroke using MERLIN, a robotic system based on serious games for upper limb rehabilitation in the home setting
Source: J Neuroeng Rehabil. 2021 Feb 23;18:41. doi: 10.1186/s12984-021-00837-z (PMC7901008; doi:10.1186/s12984-021-00837-z)
Supplement: Supplementary file 1 — Additional file 1. Intrinsic Motivation Inventory (IMI). [file 12984_2021_837_MOESM1_ESM.docx]

ADAPTED INTRINSIC MOTIVATION INVENTORY

For each of the following statements, please indicate how true it is for you, using the following scale:

| 1 | 2 | 3 | | 4 | 5 | | 6 | 7 |
| --- | --- | --- | --- | --- | --- | --- | --- | --- |
| Not at all true | | | Somewhat true | | | Very true | | |
|  | | |  | | |  | | |

| **Questions** | **1** | **2** | **3** | **4** | **5** | **6** | **7** |
| --- | --- | --- | --- | --- | --- | --- | --- |
| 1. I enjoyed doing this activity very much. |  |  |  |  |  |  |  |
| 2. I think I am pretty good at this activity. |  |  |  |  |  |  |  |
| 3. I put a lot of effort into this. |  |  |  |  |  |  |  |
| 4. I did not feel nervous at all while doing this. |  |  |  |  |  |  |  |
| 5. I thought this was a boring activity. (R) |  |  |  |  |  |  |  |
| 6. I believe this activity could be of some value to me. |  |  |  |  |  |  |  |
| 7. I believe I had some choice about doing this activity. |  |  |  |  |  |  |  |
| 8. I didn't try very hard to do well at this activity. (R) |  |  |  |  |  |  |  |
| 9. I think that doing this activity is useful for *my upper limb* |  |  |  |  |  |  |  |
| 10. This activity did not hold my attention at all.(R) |  |  |  |  |  |  |  |
| 11. I felt very tense while doing this activity. |  |  |  |  |  |  |  |
| 12. I felt like it was not my own choice to do this task. (R) |  |  |  |  |  |  |  |
| 13. I thought this activity was quite enjoyable |  |  |  |  |  |  |  |
| 14. I would be willing to do this again because it has some value to me. |  |  |  |  |  |  |  |
| 15. I am satisfied with my performance at this task. |  |  |  |  |  |  |  |
| 16. It was important to me to do well at this task. |  |  |  |  |  |  |  |
| 17. I did this activity because I wanted to. |  |  |  |  |  |  |  |
| 18. This was an activity that I couldn’t do very well. (R) |  |  |  |  |  |  |  |
| 19. I felt pressured while doing these. |  |  |  |  |  |  |  |
| 20. I think doing this activity could help me *to recover agility in my movements.* |  |  |  |  |  |  |  |
